# Amyotrophic Lateral Sclerosis and Metabolomics: Clinical Implication and Therapeutic Approach

**DOI:** 10.1155/2013/538765

**Published:** 2013-03-14

**Authors:** Alok Kumar, Devlina Ghosh, R. L. Singh

**Affiliations:** ^1^Center for Shock, Trauma and Anesthesiology Research (STAR) and the Department of Anesthesiology, School of Medicine, University of Maryland, Baltimore, MD 21201, USA; ^2^Department of Pharmacology and Molecular Sciences, The Johns Hopkins University School of Medicine, 733 N. Broadway, Baltimore, MD 21205, USA; ^3^Department of Biochemistry, Dr. Ram Manohar Lohia Avadh University, Faizabad 224001, India

## Abstract

Amyotrophic lateral sclerosis (ALS) is one of the most common motor neurodegenerative disorders, primarily affecting upper and lower motor neurons in the brain, brainstem, and spinal cord, resulting in paralysis due to muscle weakness and atrophy. The majority of patients die within 3–5 years of symptom onset as a consequence of respiratory failure. Due to relatively fast progression of the disease, early diagnosis is essential. Metabolomics offer a unique opportunity to understand the spatiotemporal metabolic crosstalks through the assessment of body fluids and tissue. So far, one of the most challenging issues related to ALS is to understand the variation of metabolites in body fluids and CNS with the progression of disease. In this paper we will review the changes in metabolic profile in response to disease progression condition and also see the therapeutic implication of various drugs in ALS patients.

## 1. Introduction

Motor neuron diseases (MND) are a heterogeneous group of disorders which result in death of motor neurons. These diseases may give rise to characteristic perturbations of the metabolome. Amyotrophic lateral sclerosis (ALS) is the most common form of MND in adults, affecting both anterior horn cells and corticospinal tracts. “Amyotrophic” refers to the muscle atrophy, weakness, and fasciculation that signify disease of the motor neurons. The median age of ALS onset is 55 years. Fifty percent of patients die within three years of onset of symptoms, and 90% die within five years [1]. The incidence of ALS is from approximately 2 per 100,000 per year [2] and may be increasing [3]. The lifetime risk of ALS is 1 in 600 to 1 in 1000. The majority of ALS cases which have been reported are sporadic (SALS); 10% are familial (FALS), some of which arise from mutations in superoxide dismutase-1 (SOD1) [4], TAR DNA-binding protein (TDP43) [5, 6] and fused in sarcoma/translated in liposarcoma (FUS/TLS) [7, 8], ALS2 [9, 10], dynactin [11], and senataxin [12]. Genomic studies suggest the existence of at least eleven additional loci for FALS, but the genetic defects remain to be identified [13]. Using a genome-wide association study (GWAs) approach, it has been recently reported that a locus on chromosome 9p21 accounted for 40% of familial ALS and nearly 1 fourth of all ALS cases in a sample of 405 Finnish patients [14]. This association signal had previously been reported by van Es et al. [15] as related to ALS, and a meta-analysis amongst many studies showed that this was indeed the major signal for this disease [16]. Similarly, recent GWAs for frontotemporal dementia (FTD) with TDP-43 pathology had also been identified on this locus [17]. Linkage analysis of patients affected with multiple cases of ALS, FTD, and FTD-ALS with type 2 TDP-43 pathology had suggested that there was an important locus for the disease on chromosome 9p [18–21], but it is not clear whether the linkage and association signals related to a single locus or whether the different studies are reporting the same alleles at that locus. Some more studies have shown that there is a large hexanucleotide (GGGGCC) repeat expansion in the first intron of C9ORF72 on the affected haplotype [22], and a common Mendelian genetic lesion in C9orf72 is implicated in many cases of sporadic and familial ALS and FTD [23]. Recently two important discoveries have been made in ALS genetics. One of them is mutations in UBQLN2, which encodes a ubiquitin-like protein, ubiquilin 2, cause dominantly inherited chromosome X-linked ALS and ALS/dementia [24]. Ubiquilin 2 is a member of the ubiquilin family (ubiquilins), which regulate the degradation of ubiquitinated proteins and found in both inherited and sporadic form of ALS. Functional analysis showed that mutations in UBQLN2 lead to an impairment of protein degradation, abnormal protein aggregation and neurodegeneration. Another is mutation in SQSTM1 gene, which encodes the ubiquitin-binding protein p62 (also known as sequestosome-1). These findings provide evidence of a direct genetic role of p62 in ALS pathogenesis as it is supposed to be involved in protein-recycling system by regulating the protein degradation pathways [25], and thus, P62, related to UBQLN2 may represent an important therapeutic target in ALS. The key role these mutations play in ALS points towards their probable contribution in ALS metabolomics, which needs to be explored in future research studies.

Although the root cause of ALS is not clearly understood, but multiple mechanisms have been found to be associated in the pathogenesis of motor neuron death in ALS. These include oxidative stress, glutamate-mediated excitotoxicity, protein aggregation, mitochondrial dysfunction, and transition-metal-induced toxicity [26–28].

## 2. Metabolomics

Metabolomics is the comprehensive study of the repertoire of small molecules present in cells, tissues, or other biological samples [29]. The lack of biological tools to detect ALS together with the nonspecificity and heterogeneity of clinical symptoms leads to difficulty in diagnosing the disease in its early stages. Metabolomic analysis is a universally applicable strategy for defining the metabolite composition of cells and tissues. Ideally metabolomic analyses are rapid, unbiased, and comprehensive, and, to some extent mass spectrometry, H1 nuclear magnetic resonance (H1 NMR) spectroscopy and IR spectroscopy are progressively considered as metabolomic techniques. 

Metabolomic approach evolves to study most or all implicated pathways, thereby revealing a biochemical signature for the disease and providing valuable new insights into disease mechanisms. Metabolomics should be seen as a complementary technique to genomics, transcriptomics, and proteomics. Additionally, it will be of great interest to integrate metabolomic, transcriptomic, and proteomic data into a system biology approach to understand global changes in ALS biological states. Also metabolomics may prove to be a powerful tool in early digonosis of ALS. The understanding of regulatory metabolic processes of a complex living organism at the system level requires the assessment of spatiotemporal interorgan metabolic crosstalks through the analysis of biofluids. This challenge can be addressed by studying the metabolic correspondence among tissue and biofluid metabolic profiles. When associated with a well-defined physiological condition, metabolic profiles provide a snapshot of a functional phenotype, or metabotypes, as a result of multiple interactions between metabolic pathways under the influence of environment, lifestyle, genetics, and microbial factors [30–32]. A specific metabolic profile of a systemic biofluid, such as blood or serum/plasma, reflects the overall metabolic status of an individual as the result of highly complex metabolic exchanges between diverse biological compartments, including organs, biofluids, and microbial symbionts. Although H1-NMR is a well-established approach for multicompartmental metabolic profiling of intact tissue and biofluid samples [33], GC/TOFMS has the advantage to discover changes in metabolites present at lower concentrations due to the increased sensitivity; hence, NMR and mass spectrometry should be regarded as complementary techniques.

## 3. ALS and Peripheral Biomarkers

The potential use of blood (serum or plasma) and cerebrospinal fluid (CSF) biomarkers associated with ALS have been studied by many authors, and these target molecules are supposed to be linked to ALS pathogenesis [34–36]. Recently, high-throughput techniques have been used to evaluate a combination of markers in patients with neurological diseases [37] which is performed via different analytical methods such as high-performance liquid chromatography followed by electrochemical detection [38].

### 3.1. Blood Biomarkers

**Figure 1:**
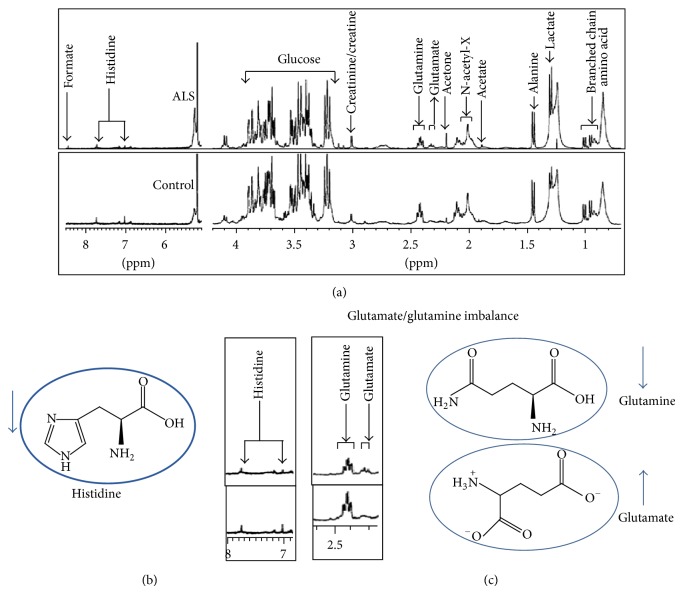
(a) Representative serum NMR spectra of control subjects and ALS patients, (b) and (c) show imbalance between oxidative/antioxidant and glutamate/gluatamine cycle. It has been speculated that glutamate excitotoxicty exacerbates the formation of ROS, which may be responsible for the oxidant-antioxidant imbalance, such as increased BHBT and acetone and decreased concentrations of histidine [26].

NMR spectroscopy is a noninvasive technique, needs little sample preparation, and gives an overview of the principal metabolic pathways. Recently, Kumar et al. have analyzed blood (serum) metabolic profile to study 13 metabolites by H1 NMR (Figure 1) [39] and found a significant elevation of the metabolite glutamate in serum sample of ALS patients. These higher glutamate signals were consistent with the hypothesis of glutamate excitotoxicity in ALS pathogenesis. Plaitakis [40] proposed the hypothesis of glutamatergic dysfunction in ALS, that is, imbalance between brain and blood glutamate levels. Babu et al. also observed that blood glutamate levels were significantly higher in ALS patients [41]. Furthermore, Kumar et al. found that, with increased glutamate concentration, glutamine concentration decreased in ALS, which might represent the imbalance between glutamate-glutamine conversion cycle that occurs in postsynaptic buttons and astrocytes during excitotoxicity [39]. Earlier study of Pioro [42] also showed in vivo evidence of abnormal glutamate metabolism in the CNS parenchyma of ALS patients.

There were previous studies by authors, who measured glutamate and found decreased glutamate concentrations in CNS tissue and increased concentrations in the serum and CSF of ALS patients, and hence, proposed a hypothesis suggesting an imbalance in the intracellular versus extracellular glutamatergic neurotransmitter system [43, 44]. Further some more important metabolites related to energy and oxidant and antioxidants have been studied, and it has been hypothesized that glutamate excitotoxicity exacerbates the formation of ROS, which may be responsible for the oxidant-antioxidant imbalance in serum of ALS patients. For example, histidine metabolite, which is considered to be powerful antioxidant, and N-acetyl derivative (N-acetyl-X) concentrations significantly decreased in Serum of ALS patients with increase concentration of BHBT, acetone, and glutamate (Figure 1) [45]. 

Unfortunately due to space limitations only a small number of promising article findings related to blood metabolites can be discussed in this section, and readers are directed to other excellent research articles for some other changes in metabolic profile in blood of ALS patients [45–47].

### 3.2. CSF Biomarkers

CSF is known as good source for the study of biomarkers because of its direct contact with the brain, its accessibility, and its dynamic changes with the cerebral environment.

Reduced levels of cystatin C and increased levels of neurofilaments have been proposed as CSF biomarkers for ALS [48, 49]. A recent study has found a relationship between the levels of Galectin-3 to be indicative of the onset of ALS symptoms in mice, and the result was found transferrable to ALS [50]. Blasco et al. [51] measured 17 metabolites in ALS CSF and compared CSF metabolic profiles between ALS and non-ALS patients. His univariate analysis showed higher concentrations of CSF acetone, pyruvate, and ascorbate in ALS patients, while acetate was found decreased in ALS compared to controls. In this interesting study, only a small part of the metabolome was studied. The authors concluded that perturbations in energy metabolism and ketone metabolism were associated with ALS. However, other earlier studies of ascorbate metabolism have shown contradictory results [52, 53]. The elevated levels of this antioxidant molecule (ascorbate) are compatible with oxidative stress previously described in ALS and could also be linked to ascorbic acid release from astrocytes after glutamate stimulation [54, 55]. In earlier studies performed over the past twenty years and using different methodologies and usually only a small number of patient samples, glutamic acid has been reported as being elevated, normal, or reduced in the CSF of ALS patients [56–58]. The studies reporting an increase in CSF glutamic acid content have been taken as support for the theory of glutamic acid excitotoxicity as a cause of ALS [56] and could result in elevated levels of ascorbic acid. Ascorbic acid might, by itself, modulate neuronal metabolism through the inhibition of glucose consumption during episodes of glutamatergic synaptic activity and by stimulating lactate uptake in neurons [59]. In contrast to the interesting study by Blasco et al. [51], Wuolikainen et al. [60] found that the content of ascorbic acid to be nonsignificantly different between ALS and controls. Creatinine was reduced in the ALS patients. The sources of the compound, creatine, and creatine phosphate, are important for the energy metabolism and are present at high levels in the CNS. The major formation of creatinine in the body, however, takes place in the skeletal muscles. CSF creatinine levels reflect both local production of the compound and plasma creatinine levels [61]. This suggests that amyotrophy is the most likely explanation to the lower CSF creatinine levels in ALS. Wuolikainen et al. [60] studied the CSF metabolome by using GC-TOFMS platform in ALS patients with six different mutations in the SOD1 gene and compared it with ALS-patients without such mutations and found that patients with a SOD1 mutation have a distinct metabolic profile in the CSF. In particular, the patients who are homozygous for the D90A SOD1 mutation showed a distinctively different signature when modeled against ALS patients with other SOD1 mutations and sporadic and familial ALS patients without a SOD1 gene mutation. Among the metabolites that contributed most to the CSF signature were arginine, lysine, ornithine, serine, threonine, and pyroglutamic acid, all found to be reduced in patients carrying a D90A SOD1 mutation. Analysis of the neurofilament light chain in the CSF suggests that patients with SOD1 gene mutations constitute a distinct subgroup within the ALS syndrome, and more so patients homozygous for the D90A SOD1 mutation [49].

Wuolikainen et al. [60] also reported that patients with SALS have a heterogeneous metabolite signature in the CSF. However, FALS without SOD1 mutation is less heterogeneous than SALS. The metabolome of the CSF of ALS patients with a SOD1 gene mutation was found to form a separate homogeneous group. Analysis of metabolites revealed that glutamate and glutamine were reduced, particularly in patients with a familial predisposition. There are significant differences in the metabolite profile and composition among patients with FALS, SALS, and patients carrying a mutation in the SOD1 gene suggesting that the neurodegenerative process in different subtypes of ALS may be partially dissimilar.

### 3.3. Kynurenine Pathway and Its Metabolites in Blood and CSF

**Figure 2:**
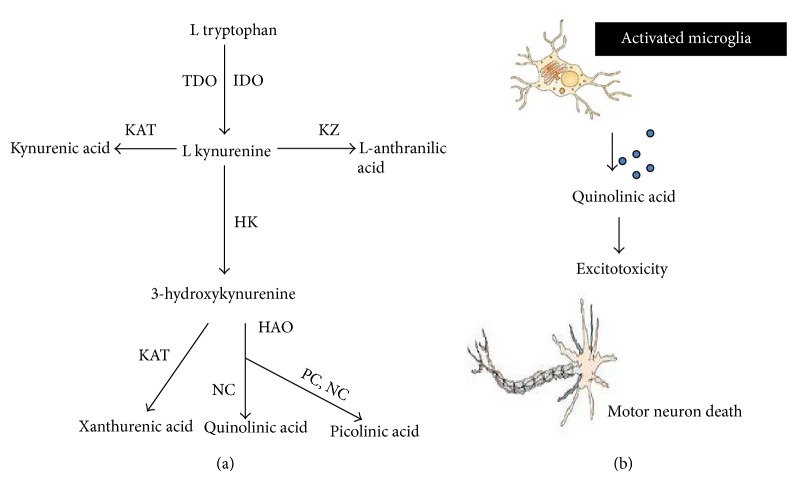
(a) Schematic diagram of kynurenine pathway. TDO—tryptophan 2,3-dioxygenase, IDO—indoleamine 2,3-dioxygenase, KAT—kynurenine aminotransferase, KZ—kynureninase, HK—kynurenine 3-hydroxylase, HAO—3-hydroxyanthranilate-3,4-dioxygenase, PC—picolinic carboxylase, NC—nonenzymic cyclization. Details are given in the text. (b) Schematic representation of QUIN toxicity in motor neurons. Due to presence of large number of activated glial cells in ALS, there is release of large amount of QUIN into the microenvironment, which can then result in excitotoxicity in motor neurons via NMDA receptors, or it can also be taken up in large quantities by motor neurons. Ultimately, excitotoxicity and the accumulation of QUIN contribute to the demise of motor neurons.

Kynurenine pathway (KP) plays a very important role in the pathogenesis of ALS. The KN is a major route for the conversion of L-tryptophan (TRP) to nicotinamide adenine dinucleotide (NAD) [62, 63], initiated by either tryptophan 2,3-dioxygenase (TDO) or indoleamine 2,3-dioxygenase (IDO) [64, 65]. Along the kynurenine (KN), neuroactive kynurenine intermediates are produced. They include the free radical generator 3-hydroxykynurenine (3-HK) [66], the excitotoxic N-methyl-D-aspartate (NMDA) receptor agonist, quinolinic acid (QUIN) [67], the neuroprotective NMDA receptor antagonist, kynurenic acid (KYNA) [68, 69], and the neuroprotective picolinic acid (PIC) (Figure 2) [70, 71]. With respect to the pathogenesis of ALS, there is increasing evidence suggesting the involvement of the KP [72], especially that of the neurotoxin QUIN. In familial SOD1 ALS, the formation of covalent aggregates of SOD1 is tryptophan dependent [73]. Kynurenic acid (KYNA), produced from l-kynurenine, is an endogenous antagonist of ionotropic excitatory amino acid receptors acting preferentially at the glycine site associated with the NMDA receptor complex [4]. Turski et al. [74] demonstrated the highest KYNA levels in the caudate nucleus, lower levels in the thalamus, globus pallidus, hippocampus, parietal cortex and frontal cortex, and the lowest level in the cerebellum. It has been suggested that the reduced concentration of KYNA may cause neurodegeneration [75]. On the other hand, this endogenous neuroprotectant could have beneficial effect on several animal models of neurological diseases [62, 63]. Iłzecka et al. [76] demonstrated that KYNA concentration was significantly higher in the CSF of ALS patients with bulbar onset of ALS compared to control group, whereas serum KYNA concentration was significantly lower in ALS patients with severe clinical status than in controls. These findings suggest that in ALS patients CSF KYNA concentration does not depend on its production in the periphery. But recent study by Chen et al. [77] in ALS patients has shown twofold increase in CSF TRP, serum TRP, and serum KYN, a tenfold increase in CSF KYN and a fourfold increase in intracellular CSF IDO activity in ALS compared to controls. Although KYN can be transported across the blood brain barrier, the tenfold increase in CSF KYN, compared to only a twofold increase in serum KYN, is strongly indicative of a CNS source. The elevated levels of serum and CSF TRP are speculated to reflect a dysfunction in protein-binding and L-neutral amino acid transporters, respectively, or perhaps, an over-compensatory response, whereby the increase in CSF IDO activity results in a decrease in CSF TRP, which encourages the dissociation of TRP from albumin to facilitate transport of TRP across the blood brain barrier (BBB). A recently published study with glutamate has found that abnormal neurons exhibit an upregulation in a particular form of glutamate-aspartate transporter, which may make them more vulnerable to glutamate-mediated excitotoxicity [78]. As such, a similar event is hypothesized to occur between QUIN and motor neurons. Pathological concentrations of QUIN could result in a myriad of unfavourable consequences that could exacerbate and accelerate the condition of ALS. As an endogenous NMDA agonist, QUIN is a potent excitatory compound [67]. In the brain, the main source of QUIN is immune cells rather than astrocytes or neurons [79, 80]. Indeed, astrocytes lack the enzyme kynurenine hydroxylase and are incapable of synthesizing QUIN [79]. However, high levels of KYN produced by astrocytes can be taken up by surrounding activated microglia for QUIN production [79]. Once QUIN exceeds the “safety threshold” (<150 nM), its adverse effect is exerted via the generation of reactive oxygen species [81], augmentation of the excitotoxic impact through disruption in the glutamatergic transport system, apoptosis, mitochondrial dysfunction, and the production of cytokines and chemokines, all of which are putatively thought to be contributory factors in ALS (Figure 1) [72]. Chen et al. [77] showed a significantly increased microglial activation in ALS motor cortex and spinal cord which indicate a heightened immune response in ALS CNS. It has been also shown that synthesis of KYNA in the brain takes place predominantly in astrocytes [82]. In ALS, an intensive astrocytosis has been found. Schiffer et al. [83] reported reactive astrogliosis in the ventral horns, dorsal horns, and at the transition between gray matter and anterior and lateral funiculi in the spinal cords of ALS patients. Feeney et al. [84] showed that motor neuron loss and reactive astrocytosis were correlated with the progression of the disease in SOD 1 transgenic mouse model of ALS. Bendotti et al. [85] observed that the loss of glutamate transporter subtype 1 (GLT-1) in ALS transgenic SOD1 mice selectively occurs in the areas affected by neurodegeneration and reactive astrocytosis. Thus, it seems that astrocytosis occurring in ALS may cause an increase of KYNA production and observed elevation of KYNA in CSF.

## 4. Brain Metabolites

ALS is a neurodegenerative disease characterized by progressive degeneration of both upper motor neurons (UMN) in the primary motor cortex (PMC) and lower motor neurons (LMN) in the brain stem and spinal cord anterior horns. Despite the identification of pathologies in the PMC and corticospinal tracts (CST) of autopsy tissue samples with ALS [84–87], there are no biomarkers identified to date that reliably indicate presence of such pathologies in the brain of patients with ALS. In an effort to find neuroimaging biomarkers indicative of UMN degeneration, advanced MR techniques (e.g., magnetic resonance spectroscopy, diffusion tensor imaging, and functional MRI) have been explored [88, 89]. It is hypothesized that the neuropathologically proven motor neuron and CST degeneration in ALS might occur gradually with no apparent manifestation of macroscopic tissue structural changes in the early stage which are detectable by conventional MRI methods. In contrast, neurochemicals indicative of metabolic processes responsible for degeneration of the motor neurons and CST in patients with ALS can be accessed from the disease onset stage using proton MR spectroscopic (1H MRS) methods. Several previous cross-sectional and longitudinal studies have demonstrated the value of proton MRS for the evaluation of metabolite alterations in the PMC [90–100] and CST [95, 101] of patients with ALS using single voxel SVS and 2D MR spectroscopic imaging [101–103] methods. In general, most of these studies have reported significantly altered metabolite concentrations or ratios in the motor pathways of patients with ALS.

1H MRS of the human brain reveals important information about compounds such as N-acetylaspartate (NAA) which has been suggested to be a marker for neuron viability and axonal density in the brain [104], creatine (Cr) as a putative marker of gliosis [105, 106], and choline (Cho) which is associated with membrane phospholipids [107]. Several 1H MRS studies in patients with definite ALS and clear signs of UMN involvement showed a reduction of NAA in the motor cortex [90, 108–110]. In another study, chemical shift-imaging follow-up measurements in ALS patients after a three-month period demonstrated a decline in concentrations of NAA, Cr, and Cho in the most affected motor cortex areas of ALS in comparison with healthy controls [111]. Unrath et al. [97] found significant changes of NAA concentrations and the NAA/(Cr + Cho) ratio of the investigated grey matter areas. It has been observed that the ratio of NAA and NAA/(Cr + Cho) as a biomarker of neuronal loss or neuronal metabolism in ALS patients is significantly apparent. This was supported by significantly lower NAA values in the more affected hemispheres at the time of study inclusion. Kalra et al. [93] also reported significantly decreased NAA/*myo* inositol in ALS patients compared to healthy controls. 

## 5. Muscle Metabolite

Today ALS is thought to be to some degree a multisystem disorder and mitochondrial abnormalities that have been observed not just in motor neurons but even in skeletal muscle cells of ALS patients. The mechanisms underlying the selective motor neuron degeneration in ALS remain elusive. Recent work in mSOD1 mice has shown that motor neuron death is not cell autonomous and involves defects in other cell types than neurons [112, 113]. Consistent with this notion, recent evidence showed that the pathophysiology of ALS includes widely systemic defects in both patients and animal models. In particular, energy homeostasis is strikingly abnormal in ALS patients since both SALS and FALS patients present with increased energy expenditure (hypermetabolism) and hyperlipidemia [114, 115]. Further it is also reported that, a severe deficiency of nicotinamide adenine dinucleotide to CoQ oxidoreductase (NAOH: CoQ) in skeletal muscle biopsies of patients with SALS was observed using saponin-permeabilized muscle fibers [116]. This muscular mitochondrial defect had a heterogeneous distribution [117] and was correlated in some patients with multiple mitochondrial DNA deletions, decreased mitochondrial DNA levels, and low levels of membrane-associated manganese SOD [118]. These results suggest that some SALS patients may have muscular mitochondrial damage that may contribute to disease pathogenesis. It has been found that metabolism is higher and bodyweight and fat mass are lower in mutant SOD1 mice than in wild-type mice [119]. These signs occur weeks before disease onset [119]. Thus, muscle hypermetabolism and energy deficit are intrinsic to ALS pathogenesis. Findings from newly generated TDP-43 animal models also imply defective energy homoeostasis in ALS. Loss of TDP-43 [120] or its overexpression [121, 122] both lead to growth retardation and thus impaired energy homoeostasis. Furthermore, adult loss of TDP-43 led to massive decreases in adipose tissue, probably through muscle hypermetabolism [120], and TDP-43 overexpression led to the formation of morphologically abnormal mitochondria [121, 122]. TDP-43-ALS, like mutant SOD1-ALS, therefore, seems to be associated with impaired energy homoeostasis in transgenic animals. The mechanisms linking TDP-43 and mitochondrial physiology needs further investigation. Abnormalities in muscle energy metabolism have been suggested as the direct cause of energy deficit and hypermetabolism in mutant SOD1 mice. Cellular levels of ATP are decreased [122–124] and expression of mitochondrial uncoupling proteins and concentrations of markers of lipid and carbohydrate use are increased [120, 125]. Several reports have indicated the existence of mitochondrial defects in the muscle tissue of patients with ALS that develops as the disease progresses [126–128]. Low-level mitochondrial defects might, however, be present earlier, as increased sensitivity of ALS myoblasts to oxidative stress has been seen in some patients [129]. Localisation of mitochondrial defects to regions close to neuromuscular junctions has also been suggested [130]. The extent and origin of such mitochondrial dysfunction remain controversial, however, and the impairment of energy metabolism seen in patients with ALS and mutant SOD1 mice might be due at least partly to dysfunctional regulation of metabolic pathways. This effect might be potentiated by the cooccurrence of a mitochondrial defect. Decreases in the efficiency of muscle energy metabolism lead to motor neuron degeneration. 

## 6. Urinary Collagen Metabolite

Numerous studies in ALS patients have shown collagen abnormalities of skin, such as decreased amount of collagen [131], alteration of cross-linking of collagen [132], and increased solubility of collagen [133]. Recently, it has been demonstrated that the basement membrane of skin in ALS patients is weakly positive for type IV collagen compared with controls, and also serum type IV collagen levels in ALS patients are lower than controls [134]. Increase in urinary excretion of collagen metabolites detects the degradation of collagen [135]. It showed that the absolute and relative concentrations of two collagen metabolites, glucosylgalactosyl hydroxylysine (glu-gal Hyl), and galactosyl hydroxylysine (gal Hyl), excreted in urine, indicate the tissue origin of the collagen metabolites and the rate of degradation of collagen [135–137]. Glu-gal Hyl accounts for 61% of the hydroxylysine glycosides in skin collagen, and gal Hyl constitutes approximately 75% of the glycosides in bone collagen [135, 138]. Measurement of glu-gal Hyl and gal Hyl may indicate whether bone or skin collagen is preferentially being degraded [135]. The study demonstrated that large increase in degradation of skin collagen produces a large increase in urinary excretion of glu-gal Hyl [139]. It has also been reported that, in the case of bone collagen, patients with bone complications tend to have elevated urinary gal Hyl excretion [140]. A high ratio of glu-gal Hyl to gal Hyl increases the probability that the patient will develop skin complications, and a low ratio is associated with bone-related complications [135]. Therefore, these two hydroxylysines, that is, glu-gal Hyl and gal Hyl, offer more reliable parameters of collagen breakdown than hydroxyproline [140]. The present study has indicated that the urinary excretion of glu-gal Hyl is markedly decreased in ALS and is more pronounced with the duration of illness. Ono et al. [141, 142] reported markedly decreased contents of collagen and collagen-associated amino acids in the lateral corticospinal tract and the anterior horn in the spinal cord of patients with ALS, suggesting that these data could be associated with the degeneration of the upper and lower motor neurons in patients with ALS.

## 7. Therapeutic Consequences

### 7.1. Riluzole (2-Amino-6-Trifluoromethoxy Benzothiazole)

Riluzole was the first drug approved by the FDA (USA) in 1995 for the treatment of ALS, but its mechanisms of action in slowing the progression of this disease remain obscure. In initial study, to evaluate the efficacy and safety of the antiglutamate agent riluzole, Bensimon et al. [143] conducted a prospective, double-blind, placebocontrolled trial in 155 ALS patients and found that the antiglutamate agent riluzole appears to slow the progression of ALS disease, and it may improve survival in patients with bulbar onset of disease. Further, Lacomblez et al. [144] also carried out a double-blind, placebocontrolled at multicentre and found that the 100 mg dose of riluzole has the best benefit-to-risk ratio, and it decreased mortality and slowed muscle-strength deterioration in ALS patients. It has been reported that the drug riluzole induces a strong metabolic signature which seems not to derive chemically from the drug, but may reflect modified metabolic processes. Elucidation of the structures of these molecules could identify biochemical pathways that are subject to perturbations by the drug, some of which could contribute to its efficacy and some to its side effects [38]. Though the precise mechanism of action for riluzole remains unclear, it appears to interfere with excitatory amino acid signalling, possibly through the inhibition of glutamate release [145–147], blockade of inactivated sodium channels [148], and interaction with guanosine triphosphate-binding proteins [149].

### 7.2. Minocycline

minocycline is approved by the FDA (USA) for bacterial infection treatment [150]. It is a tetracycline with anti-inflammatory action coupled with an independent antimicrobial property. It effectively penetrates the BBB and is clinically well tolerated. minocycline has proved to be a promising neuroprotective agent [151–154] when studied in mouse models of cerebral ischemia, spinal cord injury, and Parkinson's disease (PD). As an anti-inflammatory, minocycline inhibits apoptosis (programmed cell death) via the attenuation of TNF-*α* and downregulating proinflammatory cytokine output. This effect is mediated by a direct action of minocycline on the activated T cells and on microglia, which results in the decreased ability of T cells to contact microglia which impairs cytokine production in T-cell-microglia signal transduction [155]. It has been also found that minocycline inhibits mitochondrial permeability-transition-mediated cytochrome-c release [156]. A pilot study on ALS patients showed that it could be safe to take minocycline together with riluzole with no significant side effects [157]. In a recently completed multicentre randomized placebocontrolled phase III trial, however, the positive outcome seen in ALS mouse models was not reproduced in the human study. Instead, minocycline proved deleterious, hastening the decline of ALS patients [158]. 

### 7.3. Combination Drug Therapy (Minocycline, Riluzole, and Nimodipine)

It has been reported that minocycline, which is known as an antibiotic and inhibitor of microglial activation, riluzole, which works as an inhibitor of glutamate release, and Nimodipine, which is voltage-gated calcium channel blocker, is designed to target the different pathways leading to neuronal death [159]. Formerly, minocycline and riluzole have each alone been effective in delaying progression of ALS [143, 144, 156, 160, 161]. When the three drugs were tested in combination on SOD1 mice, it resulted in strikingly improved conditions, delaying disease onset by 4 weeks, increasing survival by 6 weeks, and attenuating neurodegeneration by reducing cyclin-dependent kinase 5 mislocalization, caspase-3 activation, astrocytosis, and microgliosis [159].

### 7.4. Ceftriaxone

Ceftriaxone is known as effective lactam antibiotics. It has been found that in rat model it has the capability to increase GLT1 expression by threefold [162]. It has been also reported that Ceftriaxone considerably reduced motor neuron loss and hypercellular gliosis, delaying muscle strength and body weight loss and improving longevity in SOD1G93A mice [162].

### 7.5. Antioxidants

Various antioxidants have been used as potential therapeutic agents in transgenic mice expressing the mutated human SOD-1 enzyme. Polyamine or putrescine-modified catalase, an antioxidant enzyme that removes hydrogen peroxide and has good permeability at the BBB, increases the survival of transgenic mice bearing the human mSOD-1G93A [147, 148, 163, 164]. Moreover, the copper chelator and thiol compound penicillamine, the copper chelator trientine, carboxyfullerenes, vitamin E, and N-acetylcysteine have been reported to increase the survival time in this mouse model and/or delay the onset of the disease to a small extent [165, 166].

### 7.6. Advantages of a High-Energy Diet in ALS

In ALS, weight loss is frequently observed and can occur early or later during the course of disease. All patients lose some weight due to the unavoidable reduction in skeletal muscle mass that results from denervation and decreased physical activity [167]. In many cases, however, weight loss also has a nutritional component, with loss of fat mass and fat-free mass (FFM) attributable to malnutrition, the principal cause of which is decreased dietary intake [168, 169]. Swallowing disorders and dysphagia affect most ALS patients but occur earliest in bulbar-onset form. A reduction of energy intake is associated with increased weight loss and the degree of dysphagia [170]. Anorexia, digestive disorders, and upper extremity motor difficulties also contribute to low intake. Another cause of malnutrition is an increase in energy requirements sufficient to exceed intake. Reports that resting energy expenditure (REE) may be increased in this context [171, 172] appear paradoxical because FFM, the main determinant of REE, decreases in ALS [4]. 

Many research studies point towards significant role of mitochondria in ALS pathogenesis [173] and thus may provide a cause of malnutrition in ALS patients. It has been found that prevalence of substantial mitochondrial degeneration in motor neurons of G93A SOD1 mutant mice at the onset of the disease [174], when electron transfer chain activity and ATP synthesis appear severely dysfunctional [175]. Furthermore, reduction in the activity of cytochrome-c oxidase, encoded by the mitochondrial genome, in motor neurons of SALS patients has also been reported [176]. There is a direct correlation between the amount of mitochondrial DNA in the spinal cord of ALS patients with decrease in the activities of citrate synthase and the respiratory chain complexes I, II, III, and IV [177]. Not only nervous system but other tissues, including liver [178], lymphocytes [179], and muscle [180], are also affected by mitochondrial dysfunction. Wiedemann et al. [116] found a deficiency of NADH:CoQ oxidoreductase in muscles of SALS and also noticed that decreased activities of NADH:CoQ oxidoreductase and cytochrome C oxidase are associated with DNA abnormalities and reduced levels of the mitochondrial Mn-superoxide dismutase [118]. Dupuis et al. [119] reported the early increase in the mRNA levels of the mitochondrial uncoupling protein-3 in skeletal muscles of ALS-linked G86R SOD1 mutant mice. In addition, isolated mitochondria from G86R muscle tissue exhibited a reduced respiratory control ratio, which is in line with the existence of mitochondrial uncoupling [119]. Supporting the mitochondrial dysfunction hypothesis, and administration of creatine, an intracellular energy shuttle between mitochondria and sites of energy consumption that is known to ameliorate muscle function, increased the life expectancy of G93A mice [181]. Although clinical trials failed to show a beneficial effect of creatine in ALS patients [182], the findings in G93A mice invoke the existence of a characteristic energetic imbalance.

Currently, hypermetabolism in ALS remains unexplained. The suggestion that hypermetabolism can be explained by an increase in the work of respiratory muscles [183] lacks credibility because there is no correlation between REE and pulmonary function [4]. The evaluation of a patient's nutritional status and elucidation of the causes of any malnutrition are essential to provision of adequate nutritional care. In fact, malnutrition is common in ALS, with a frequency reported to be between 15 and 55%, depending on the stage of disease and the definition of malnutrition adopted [171]. Moreover, malnutrition is an independent prognostic factor for survival [171, 172]. Desport et al. [172] indicated a chronic hypermetabolism in a subset of sporadic ALS patients, which complements a decrease in fat-free mass frequently observed in these patients. Dupuis et al. [119] reported that fat-enriched high-energy diet to G86R mice resulted in an increase in body mass and adipose tissue accumulation, thus showing that increasing energy intake is sufficient to reduce the energetic deficit. Furthermore Dupuis et al. [119] also showed that the number of cells in the ventral horns of the lumbar spinal cord, which is significant lost in large sized cells, most probably representing motor neurons [184], in symptomatic G86R, mice was prevented in high-fat diet-fed animals of the same age. It is worth emphasizing, however, that at least a subset of ALS patients showed characteristic hypermetabolic phenotype [172] reminiscent of that observed in mice. From clinical perspective, the nutritional status is a prognostic factor for survival in ALS [185], and more evidence suggests that the proper customized nutritional management of patients may constitute a primary symptomatic treatment for the disease [186]. Nutritional intervention may prove to be a very significant aspect in ALS and can be explored further. 

### 7.7. Drug Related to Kynurenine Pathway (KP)

The KP is a major route for the conversion of TRP to nicotinamide adenine dinucleotide [62, 63] generating neuroactive intermediates in the process. Targeting the KP could offer a new therapeutic option to improve ALS treatment [187]. In order to proceed further in this regard, two possible approaches can be taken, either to develop analogues of the neuroprotective kynurenines or to inhibit the synthesis of the neurotoxic QUIN. Some of the below-mentioned drugs may show some promising therapeutic approach towards ALS. 

(a) Intracerebral and intraperitoneal administration of 4-chlorokynurenine, which is a precursor of 7-chlorokynurenate, with QUIN, showed successful enzymatic transamination into the active 7-chlorokynurenate, conferring neuroprotection [188, 189] by preventing QUIN neurotoxicity. (b) Laquinimod, which is a novel synthetic quinoline, inhibited disease progression and infiltration of CD4+ T-cells and macrophages into the central nervous system (CNS) [10]. (c) An immunosuppressive and anti-inflammatory prodrug, leflunomide, gets converted to its active open-ring metabolite, teriflunomide, an inhibitor of mitochondrial dihydroorotate dehydrogenase, and known to successfully reduce the development of active lesions in patients with relapsing multiple sclerosis [190]. (d) The synthesis of QUIN can also be blocked by inhibiting either kynureninase or kynurenine hydroxylase activity, thus, diverting the KP towards the synthesis of KYNA. In immune activated mice, meta-nitronemzoylalanine also significantly reduced the formation of QUIN in the blood and brain [191]. (e) Ro61-8048 is another potent kynurenine-3-mono-oxygenase (KMO) inhibitor [192]. It also reduces glutamate concentrations in the extracellular spaces of the basal ganglia in rats without impairing the learning or memory process typically associated with glutamate receptor antagonists and significantly reduces the neurotoxic levels of 3-HK and QUIN in the CNS [193]. (f) Clioquinol is a quinoline metal chelator that binds selectively to zinc and copper ions [194] with a hydrophobic nature that allows it to pass easily across the BBB. Recent research with Clioquinol in neurological disorders involving an imbalance in metal ions has led to promising results, presenting the possibility of a new therapeutic strategy [195].

## 8. Conclusion

The wide range of survival time in ALS patients suggests that multiple prognostic factors are involved; only some have been clearly identified. A specific biochemical marker for early diagnosing and for monitoring disease progression in ALS will have important clinical applications. ALS is a heterogeneous syndrome with multiple subtypes with ill-defined borders. A minority of patients carries mutations in the Cu/Zn-superoxide dismutase (SOD1) gene, but the disease mechanism remains unknown for all types of ALS. As the glutamate modulator, riluzole, is the only drug currently approved for ALS treatment, however, combination therapies that target other pathogenic mechanisms may be more effective in slowing disease progression and prolonging survival. Compounds targeting the KP offer a novel and potentially effective treatment for ALS. The most recent finding in the ALS genetics suggests a very important role of mutation in SQSTM1 and UBQLN2 genes, and it is suggested that ALS patients should be monitored in a broad spectrum to study the altered effect on metabolic pathways [24, 25]. The D90A SOD1 mutation findings suggest that metabolomic profiling using GC-TOFMS and multivariate data analysis may be a future tool for diagnosing and monitoring disease progression and may cast light on the disease mechanisms in ALS [60]. For this, the use of metabolomics as the link to pathway information in genome-wide association studies could be of great interest for mapping and interpreting the effects of different mutations and even combinations of mutations in relation to ALS subtypes [195]. We thus foresee that metabolomics will contribute to deciphering the complex interactions behind ALS. The metabolomic methodology is suitable for screening large cohorts of samples. Global metabolomics can be used for detecting changes of metabolite concentrations in samples of fluids such as CSF. Exploration of metabolomics by the use of small molecules derived from biofluids provides a strong platform to understand the metabolic characters of the living system and plays a significant role in the detection of diagnostic biomarkers. Future clinical and experimental studies, therefore, need to concentrate on the complex relations between metabolism and ALS, and in this way may answer the question of whether targeting defective metabolism in ALS is an efficient way to alter disease progression. Though a single technique is not sufficient to study the entire metabolomic profile, there is a need to converge some state of the art techniques namely, GC/MS, LC/MS, and NMR to realize the full worth of metabolomics. There is definite need to further elaborate on the sensitive and specific metabolic therapeutic signatures by advance analytical means and bioinformatics application which will help us to elucidate the structures of more signature molecules in ALS disease and provide us insight into aberrant biochemical pathways and may prove to be helpful in building up diagnostic markers and targets for drug design.

## Abbreviations

ALS: Amyotrophic lateral sclerosis
AMPA: Alpha-amino-3-hydroxy-5-methyl-4-isoxazolepropionic acid
BBB: Blood brain barrier
BHBT: D-ß-hydroxybutyrate
Cho: Choline
CNS: Central nervous system
Cr: Creatine
CSF: Cerebrospinal fluid
FALS: Familial amyotrophic lateral sclerosis
FFM: Fat-free-mass
FTD: Frontotemporal dementia
GLT1: Glutamate transporter subtype 1
IDO: Indoleamine 2,3-dioxygenase
IGF: Insulin-like growth factor
IFN: Interferon
IL: Interleukin
KMO: Kynurenine-3-mono-oxygenase
KN: Kynurenine
KP: Kynurenine pathway
KYNA: Kynurenic acid
MND: Motor neuron diseases
NAAN: N-acetylaspartate
NAD: Nicotinamide adenine dinucleotide
NMDA: N-methyl-D-aspartate
NMR: Nuclear magnetic resonance
PD: Parkinson's disease
QUIN: Quinolinic acid
PIC: Picolinic acid
SOD1: Superoxide dismutase1
TDO: Tryptophan 2,3-dioxygenase
TGF: Transforming growth factor
Th: T helper.
